# Environmental Risk Factors for Congenital Heart Disease in the Shandong Peninsula, China: A Hospital-based Case–Control Study

**DOI:** 10.2188/jea.JE20080039

**Published:** 2009-05-05

**Authors:** Shiwei Liu, Junxiu Liu, Ji Tang, Jiafen Ji, Jingwu Chen, Changyun Liu

**Affiliations:** 1 Institute of Basic Medical Sciences, Chinese Academy of Medical Sciences/School of Basic Medicine, Peking Union Medical College, Beijing, China; 2 Chinese Preventive Medicine Association, Beijing, China; 3 Pediatric Unit, Weifang Medical University Affiliated Hospital, Shandong Province, China; 4 Department of Epidemiology and Health Statistics, Weifang Medical University, Shandong Province, China

**Keywords:** heart defects, congenital, hospital-based, matched case–control study, risk factors, Shandong peninsular area of China

## Abstract

**Background:**

In China, and in Shandong province, the proportionate contribution of birth defects to infant mortality has increased, and congenital heart disease (CHD) is now the most common cause of birth defects. The cause of approximately 90% of cases of congenital heart disease is multifactorial. Little is known about modifiable environmental risk factors or regional differences. We investigated putative environmental risk factors for congenital heart disease in the Shandong province of China in order to improve prevention of CHD.

**Methods:**

We conducted a hospital-based 1:2 matched case–control study of 164 patients with congenital heart diseases and 328 controls, all of whom were retrospectively interviewed. Univariate and multivariate analyses were conducted to identify environmental risk factors for CHD.

**Results:**

The environmental risk factors associated with CHD were mother’s education level (odds ratio [OR], 0.31; 95% confidence interval [CI], 0.15–0.67), neonatal asphyxia or hypoxia (OR, 3.74; 95% CI, 1.25–11.18), number of previous pregnancies (OR, 2.68; 95% CI, 1.44–4.97), maternal upper respiratory tract infection (OR, 4.12; 95% CI, 1.56–10.85), maternal infection (OR, 7.98; 95% CI, 2.14–29.72), maternal B-mode ultrasound examination (OR, 4.05; 95% CI, 1.48–11.08), and maternal mental stress (OR, 3.93; 95% CI, 1.94–7.94) during early pregnancy. No significant interactions were observed among these factors.

**Conclusions:**

Augmenting maternal mental healthcare, obtaining regular health counseling and testing during pregnancy, preventing upper respiratory tract infections, limiting medication during early pregnancy, offering health promotion and health education to women of childbearing age (especially those with less formal education), and improving obstetric procedures and techniques may lower the occurrence of congenital heart disease.

## INTRODUCTION

The infant mortality rate has decreased due to improvements in socioeconomic, educational, and healthcare conditions, and to improvements infrastructure; however, the proportionate contribution of birth defects to infant mortality in many countries has increased,^1^ as it has in China.^2^ Congenital heart disease (CHD) is the most common birth defect, occurring in 4 to 8 per 1000 live births,^1^ and the incidence is higher if fetuses that do not survive to term are included.^3^ The prevalence is 7 to 8 per 1000 live births in China, which represents approximately 100 000 to 150 000 new cases of CHD per year.^4^ The occurrence of neural tube defects has decreased significantly because of improvements in prenatal diagnostic techniques and periconceptional folic acid supplementation.^5^ Hence, CHDs are playing a more significant role, ranking first to third among the 23 principal monitored birth defects in China,^6^^,^^7^ and in Shandong province.^8^^,^^9^ In addition, because of great improvements in medical treatment and care, some patients who would have died of CHD in the past now live into adulthood, and CHD is now being diagnosed in individuals with previously undetected disease, thus increasing the number of adults with known CHD.^10^^–^^14^ Thus, as China improves its health care system, CHD is becoming one of the most important public health problems.

In approximately 90% of CHD cases, the cause is multifactorial.^1^ These etiologies, especially inherited causes, have become better understood only in the past 10 years.^15^ Less is known about modifiable noninherited factors,^16^ and direct evidence regarding environmental exposures is limited.^17^ Moreover, there are variations in the proportional contributions of different types of CHD and regional differences in environmental risk factors.^18^^–^^20^ We conducted a hospital-based case–control design to identify possible environmental risk factors for CHD in the Shandong Peninsula of China and to provide information to assist in local prevention of CHD.

## METHODS

### Selection of participants

Cases were recruited from 3 municipal hospitals and 3 maternal and child health centers in Yantai, Weifang, and Rizhao city in the Shandong Peninsula, which is located in northeast China. All cases were younger than 7 years and had a diagnosis of, or been treated for, CHD (International Classification of Disease, 10th Revision; codes Q20-Q24) between January 2004 and January 2005. Diagnoses were confirmed by appointed senior pediatric cardiologists, based on case records.

The exclusion criteria for cases included: 1) presence of abnormalities in other organs, 2) death of birth mother, 3) divorce of birth mother, or 4) mental disorder diagnosed in birth mother, 5) inability to locate birth mother for interview, and 6) unwillingness of birth mother to be interviewed.

Two hospital-based controls were recruited to match each CHD case. The controls were recruited from inpatients, outpatients, or patients undergoing health checkups at the same medical institutions during the same period. Inpatients were preferred because their diagnoses were clear and easily obtainable. If the number of inpatients was deemed insufficient, outpatients, and then other patients, were enrolled. The matching criteria for controls were: 1) same sex, 2) age difference of less than 1 year, and 3) same geographic classification (rural or urban). A control with a diagnosis of CHD or other birth defect was excluded. Controls were also excluded for the abovementioned criteria 2)–6). When a new case was confirmed, we downloaded all information on eligible patients from the hospital information system, using the matching criteria. The 2 eligible and available controls whose time of presentation at hospital was closest to that of the case were selected.

Cases and controls were all born in Shandong province, and their birth mothers were local residents. The birth parents of participants were interviewed for this study.

### Data collection and management

In each medical institution, one senior pediatric cardiologist was appointed to identify CHD cases and patients with other birth defects, and to confirm participant eligibility. One interviewer, who was masked to the participant’s group assignment, conducted face-to-face interviews of the participant’s birth parents using a standardized questionnaire. The pediatric cardiologists and interviewers were trained uniformly before the formal interview and, to further refine the format of the questionnaire and the skills of the investigators, a preliminary trial of the interview and questionnaire was conducted with the participation of a few patients’ parents from other medical institutions. All the participants’ birth parents were retrospectively interviewed in a similar manner during the period of the study. Informed consent was obtained from each participant.

The content of the questionnaire included:

1) Demographic characteristics: child’s name, sex, birth date, address, and nationality, and parents’ education levels.
2) Conditions of participant's birth: gestational age, birth weight, fetal order, birth asphyxia or hypoxia.
3) Characteristics of birth parents: age at pregnancy, consanguineous parents, family history of inherited disease, occupational exposure history, disease history, and lifestyle (smoking status, and consumption of alcohol, tea, coffee, and cola).
4) Conditions of pregnancy: gynecologic examination results, gestational weeks until aware of pregnancy, abnormal pregnancy reactions (eg, hyperemesis gravidarum, abdominal pain, and vaginal bleeding), reproductive history, and environmental exposures (infection, medication, vaccination, cold and fever, B-mode ultrasonographic examination, and radiography, microwave ovens, recent home repair or relocation, continuous noise, and mental stress).

All data entry was completed independently by two external staves, and a database was established using Microsoft Excel 2003 after logical checking.

### Statistical analysis

Variables with data structure problems were removed by constructing frequency graphs and cross-table analysis. Univariate conditional logistic regression analysis was conducted after some research factors had been quantitatively assigned. Other transformed functions, such as *x*^2^, ln(*x*), ln(1 − *x*) or *x* ln(*x*), were all applied to each discrete ranked variable and each continuous quantitative variable to identify possible nonlinear relationships between *x* and logit *p*. Dummy variables were also created for some factors. Grouping analysis was performed on correlative variables that were statistically significant on univariate analysis. Then, based on professional judgment, we determined the candidate variables for multivariate analysis. Next, a multicollinearity diagnosis was conducted for these candidate variables, using principal component analysis. The variables were adjusted if multicollinearity was present. Finally, multivariate conditional logistic stepwise regression analysis of suspected interactive variables was performed to screen for possible risk factors for the occurrence of local CHD, and the adjusted odds ratio (OR) and 95% confidence interval (CI) were obtained. All *P* values were calculated using a 2-tailed statistical test, and *P* < 0.10 was considered statistically significant in univariate analysis. All analysis was completed using SAS statistical software, version 9.1. For the 1:2 matching conditional logistic regression analysis, the PHREG procedure was used.

### Definitions

Early pregnancy—The period from 1 month before to 3 months after insemination (early pregnancy usually refers to the first trimester of pregnancy; however, as the exact time of insemination was very difficult to ascertain, we extended this period to include the month before insemination). The time of exposure was early pregnancy, unless otherwise specified.

Grouping analysis—In order to avoid the influence of strongly correlative variables in the establishment of the multivariate regression model, we performed multivariate conditional logistic regression analysis only with 2 or more strongly correlative variables entered simultaneously in a single step. Then, the more significant variable was selected for the subsequent multivariate logistic regression.

Disease history—At least one of the following diseases in early pregnancy: cardiopathy, diabetes, hypertension, epilepsy, anemia, phenylketonuria, mental illness, nephritis, hyperthyroidism, goiter, lupus erythematosus, or hepatitis.

Occupational exposure—Employment as computer operator; radio, television, or communications technician; anesthetist; disinfection staff; nuclear medicine diagnostic doctor; nuclear physics researcher; distributor of agricultural products (fertilizers, pesticides); greenhouse, cotton, or fruit tree management; welding or smelting worker; printer; painter; dye worker; pesticide processing staff; rubber or plastics processing employee; cable manufacturing worker; or instrument manufacture or repair worker.

## RESULTS

### Characteristics of cases and controls

A total of 171 cases were recruited from inpatients. We excluded 2 (1.17%) cases of stillbirth because we lost contact with the parents, 2 (1.17%) patients whose parents were distraught and unable to participate, and 3 (1.75%) patients whose parents were unwilling to be interviewed. Therefore, a total of 164 (95.91%) cases remained for analysis. A total of 401 hospital-based controls were selected, 328 (81.80%) of whom were included in the analysis. Among this group, 278 (84.76%) were inpatients and 50 (15.24%) were outpatients. The excluded controls comprised 40 (54.79%) children who were born outside their home province or whose birth mother was not a local resident, 24 (32.88%) who were unwilling to participate in this study, and 9 (12.33%) whose birth mother had died or could not be located.

Among the 164 analyzed cases, 95 (57.93%) were boys and 69 (42.07%) were girls. The age range was 0.17 to 7 years, and the average age was 5.1 years. Among the cases, 58 (35.37%) had a ventricular septal defect, 28 (17.07%) had patent ductus arteriosus, 21 (12.80%) had tetralogy of Fallot, 15 (9.15%) had an atrial septal defect, 11 (6.71%) had pulmonary stenosis, and 31 (18.90%) had another type of CHD.

### Univariate analysis and grouping analysis

We deleted all abnormal variables that had been identified in data structure analysis. Twenty-two factors were statistically significant after univariate conditional logistic regression analysis of all 41 research factors (Cronbach’s alpha = 0.10). Function transform was conducted on gestational age, father’s smoking status, father’s alcohol consumption, father’s age and mother’s age at this pregnancy, father’s and mother’s level of education, fetal order, gestational weeks until aware of pregnancy, number of previous pregnancies, and number of spontaneous abortions, but no nonlinear function was statistically significant. Dummy variables were also created for gestational age and parents’ ages at this pregnancy, and were reassigned by logically combining the category of younger age with that of older age for a similar OR. Grouping analysis of correlative variables was conducted for father’s and mother’s level of education, father’s and mother’s age at this pregnancy, history of spontaneous abortion, number of spontaneous abortions, fetal order, and number of previous pregnancies, maternal upper respiratory tract infection (URI), maternal fever, gynecologic examination, maternal infection, and use of medication by mother. As a result of this analysis, father’s level of education, father’s age at this pregnancy, history of spontaneous abortion, fetal order, maternal fever, and gynecologic examination were removed. Sixteen candidate factors were ultimately examined in multivariate analysis. The assignment and proportion of 41 research factors among cases and controls are presented in Table 1, and the results of the univariate and grouping analyses are shown in Table 2.

**Table 1.** Risk factor exposures among cases and controls

Risk factor	Cases (*n* = 164)	Controls (*n* = 328)	Risk factor	Cases (*n* = 164)	Controls (*n* = 328)
Father’s education level			Mother’s education level		
​ master’s degree or higher	0.6*	1.5	​ master’s degree or higher	0.6	1.5
​ college	12.2	26.8	​ college	3.0	12.8
​ senior high school	25.0	31.3	​ senior high school	10.4	31.4
​ primary or junior high school	61.6	39.6	​ primary or junior high school	81.7	53.0
​ illiterate or semi-literate	0.6	0.9	​ illiterate or semi-illiterate	4.3	1.2
Gestational age			Birth weight		
​ ≥42 weeks	9.1	8.2	​ <2500 g	15.2	8.5
​ 37–42 weeks	80.5	87.2	​ ≥2500 g	84.8	91.5
​ <37 weeks	10.4	4.6			
Neonatal asphyxia or hypoxia			Gynecologic examinations		
​ yes	25.6	11.3	​ yes	39.6	28.0
​ no	74.4	88.7	​ no	60.4	72.0
Mother’s age at this pregnancy			Father’s age in this pregnancy		
​ ≥35 years	6.1	0.3	​ ≥35 years	12.2	3.1
​ 20–35 years	92.7	98.5	​ 20–35 years	87.8	95.4
​ <20 years	1.2	1.2	​ <20 years	0	1.5
Consanguineous marriage			Family inherited disease history		
​ yes	7.9	2.1	​ yes	11.6	0.9
​ no	92.1	97.9	​ no	88.4	99.1
Mother’s disease history			Father’s disease history		
​ yes	22.6	3.0	​ yes	4.3	1.5
​ no	77.4	97.0	​ no	95.7	98.5
Maternal occupational exposure			Paternal occupational exposure		
​ yes	27.4	18.9	​ yes	33.5	21.0
​ no	72.6	81.1	​ no	66.5	79.0
Maternal tea-drinking			Paternal tea-drinking		
​ yes	10.4	11.9	​ yes	36.6	28.7
​ no	89.6	86.1	​ no	63.4	71.3
Maternal coffee-drinking			Paternal coffee-drinking		
​ yes	1.8	0.6	​ yes	1.8	1.2
​ no	98.2	99.4	​ no	98.2	99.8
Maternal cola-drinking			Paternal cola-drinking		
​ yes	1.8	2.1	​ yes	0.6	1.8
​ no	98.2	97.9	​ no	99.4	98.2
Vaccination			Maternal upper respiratory infection		
​ yes	4.9	5.5	​ yes	42.7	10.1
​ no	95.1	94.5	​ no	57.3	89.9
Maternal fever			Abnormal pregnancy reaction		
​ yes	22.6	3.4	​ yes	35.4	30.8
​ no	77.4	96.6	​ no	64.6	69.2
History of spontaneous abortion			Maternal infection		
​ yes	18.3	7.0	​ yes	30.5	2.1
​ no	81.7	93.0	​ no	69.5	97.9
Maternal medication			Maternal B ultrasound examination		
​ yes	42.7	14.3	​ yes	54.3	24.4
​ no	57.3	85.7	​ no	45.7	75.6
Maternal radiography examination			Frequent use of microwave ovens		
​ yes	1.2	1.8	​ yes	1.8	0.9
​ no	98.8	98.2	​ no	98.2	99.1
New house repair or relocation			Continuous noise		
​ yes	11.6	2.4	​ yes	9.1	7.0
​ no	88.4	97.6	​ no	90.9	93.0
Mother smoked before and during the first month of pregnancy			Father smoked before and during the first month of pregnancy		
​ past smoker or never smoker	97.0	99.4	​ past smoker or never smoker	80.5	84.1
​ yes	3.0	0.6	​ yes	19.5	15.9
Mother drank alcohol before and during the first month of pregnancy			Father drank alcohol before and during the first month of pregnancy		
​ quit or never drank	100.0	99.7	​ quit or never drank	87.8	92.4
​ yes	0	0.3	​ yes	12.2	7.6
Fetal order	1.58 ± 0.72^†^	1.29 ± 0.52	Number of previous pregnancies	0.79 ± 0.86	0.35 ± 0.62
Gestational weeks until aware of pregnancy	6.49 ± 2.84	5.63 ± 2.13	Number of spontaneous abortions	0.20 ± 0.43	0.08 ± 0.29
Maternal mental stress					
​ severe	21.3	3.0			
​ mild	13.4	9.1			
​ none	65.3	87.8			

*Proportion (%).

^†^Mean ± standard deviation.

**Table 2.** Results of univariate analysis and grouping analysis

Research factors	*OR* (95% *CI*)*	Research factors	*OR* (95% *CI*)
Father’s education level^†,||^	0.53 (0.35–0.81)	Gestational weeks until aware of pregnancy^†,‡^	1.16 (1.02–1.32)
Mother’s education level^†,‡,||^	0.28 (0.15–0.54)	Vaccination (yes/no)	0.86 (0.22–3.32)
Gestational age^§^	1.78 (0.75–4.21)	Maternal upper respiratory infection (yes/no)^†,‡^	5.89 (2.65–13.07)
Birth weight (<2500 g/≥2500 g)	1.49 (0.67–3.30)	Maternal fever (yes/no)^†^	7.50 (2.49–22.60)
Fetal order^†^	1.89 (1.18–3.01)	Abnormal pregnancy reaction (yes/no)	1.23 (0.66–2.30)
Neonatal asphyxia or hypoxia (yes/no)^†,‡^	2.07 (0.91–4.72)	Number of previous pregnancies^†,‡^	2.46 (1.50–4.04)
Mother’s age at this pregnancy^†,‡,§^	7.20 (0.81–64.17)	History of spontaneous abortion (yes/no)^†^	3.06 (1.19–7.87)
Father’s age at this pregnancy^†,§^	2.62 (0.93–7.33)	Number of spontaneous abortions^†,‡^	2.49 (1.08–5.75)
Consanguineous marriage (yes/no)^†,‡^	5.00 (0.97–25.77)	Maternal infection (yes/no)^†,‡^	10.19 (2.96–35.08)
History of inherited disease in family (yes/no)**	—	Maternal medication (yes/no)^†,‡^	2.97 (1.52–5.81)
Mother’s disease history (yes/no)^†,‡^	6.65 (2.18–20.30)	Maternal B-mode ultrasound examination (yes/no)^†,‡^	1.93 (0.96–3.87)
Father’s disease history (yes/no)	6.00 (0.62–57.68)	Maternal radiography examination (yes/no)**	—
Maternal occupational exposure (yes/no)	1.52 (0.79–2.91)	Frequent use of microwave ovens (yes/no)	2.00 (0.13–31.98)
Father’s occupational exposure (yes/no)^†,‡^	1.79 (0.94–3.42)	New house repair or relocation (yes/no)^†,‡^	6.00 (1.21–29.73)
Maternal tea-drinking (yes/no)	0.85 (0.32–2.26)	Continuous noise (yes/no)	1.36 (0.47–3.98)
Paternal tea-drinking (yes/no)	1.45 (0.78–2.71)	Maternal mental stress^†,‡,¶^	2.70 (1.53–4.76)
Maternal coffee-drinking (yes/no)**	—	Paternal coffee-drinking (yes/no)	0.67 (0.07–6.41)
Maternal cola-drinking (yes/no)**	—	Paternal cola-drinking (yes/no)**	—
Mother smoked before and during the first month of pregnancy (quit or never smoked/yes)**	—	Father smoked before and during the first month of pregnancy (quit or never smoked/yes)	0.62 (0.29–1.34)
Mother drank before and during the first month of pregnancy (quit or never drank/yes)**	—	Father drank before and during the first month of pregnancy (quit or never drank/yes)^†,‡^	0.58 (0.24–0.89)
Gynecologic examinations (yes/no)^†^	2.08 (1.04–4.16)		

*Odds ratio (95% confidence interval).

^†^Statistically significant after univariate analysis with a test criterion of 0.10.

^‡^Candidate factors for multivariate analysis.

^§^Factors reassigned after function transform of the discrete ranked variables, 1: <37 or ≥42 weeks, 0: 37–42 weeks for gestational age; and 1: <20 or ≥35 years, 0: 20–35 years for mother’s and father’s age at this pregnancy.

^||^5-master’s degree or higher; 4-college; 3-senior high; 2-primary or junior high; 1-illiterate or semi-literate.

^¶^2-severe, 1-mild, 0-none.

**OR is not shown if 95% CIs are extremely wide.

### Multivariate conditional logistic stepwise analysis

Multicollinearity diagnosis showed that there was good independence within the 16 candidate variables (Kaiser-Meyer-Olkin measure = 0.55). Seven putative environmental risk factors were significantly associated with local CHD after multivariate conditional logistic stepwise regression analysis (significance level to enter = 0.05, significance level to remain = 0.10). Partial correlation analysis was performed—and combined with professional knowledge—to ascertain whether there was interaction among the 7 factors, and to identify the first-order interactive variables, such as mother’s education level × number of previous pregnancies, maternal infection × maternal URI, maternal infection × maternal B-mode ultrasound examination, maternal infection × maternal mental stress. However, we did not identify any statistically significant variables that could be explained in light of the authors’ professional knowledge.

Because the results of the PHREG procedure of the SAS software did not provide a standardized partial regression coefficient, we used the *Z* value (defined as the ratio of the partial regression coefficient to its standard error) to compare the relative importance of independent variables to dependent variables in the model: the larger the absolute value of *Z*, the greater the role of the variable. The results of multivariate analysis showed that the possible risk factors—ranked according to their influence on the occurrence of CHD, from strong to mild—were maternal mental stress, number of previous pregnancies, maternal infection, mother’s level of education, maternal URI, maternal B-mode ultrasound examination, and neonatal asphyxia or hypoxia. Detailed results are shown in Table 3.

**Table 3.** Results of multivariate conditional logistic stepwise regression analysis

Putative risk factor	OR*	95% CI*	*P*
Mother’s education level^†^	0.31	0.15–0.67	0.011
Neonatal asphyxia or hypoxia (yes/no)	3.74	1.25–11.18	0.048
Maternal upper respiratory infection (yes/no)	4.12	1.56–10.85	0.016
Number of previous pregnancies	2.68	1.44–4.97	0.009
Maternal infection (yes/no)	7.98	2.14–29.72	0.010
Maternal B-mode ultrasound examination (yes/no)	4.05	1.48–11.08	0.022
Maternal mental stress^‡^	3.93	1.94–7.94	0.001

*OR: odds ratio, 95% *CI*: 95% confidence interval.

^†^5-master’s degree or higher; 4-college; 3-senior high; 2-primary or junior high; 1-illiterate or semi-literate.

^‡^2-severe; 1-mild; 0-none.

## DISCUSSION

A hospital-based 1:2 matched case–control study was conducted to screen possible risk factors for CHD occurring in the Shandong Peninsula. Eligible cases and controls were selected according to strict criteria, and the area of coverage was specifically restricted to the Shandong peninsula. Ultimately, good results were obtained with this sample.

It is very important to use appropriate modeling strategies in studies of etiologic association. To cite one example, when linear regression analysis is performed, nonlinear relationships must be considered. It is necessary to investigate various functional relations between factors and diseases by means of variable transformation, and to consider the possibility of interactions so as to guard against the chance that potential risk factors or modes of action might be neglected. Although we observed no statistically significant transformed variable form in this study, we did gain a number of valuable insights.

We observed that maternal mental stress during early pregnancy was the most important factor (*Z* = 3.20, *P* = 0.0014) in the occurrence of CHD, and that there was a dose–response relationship between them. This association was also observed in the case–control studies of Adams et al^21^ and Carmichael et al,^22^ but those studies relied on 3 questions to identify mental stress: 1) “Did anyone close to you die?” 2) “Did you or anyone close to you lose a job?”, and 3) “Were you or was anyone close to you divorced or separated?” A positive response to one of the questions was defined as positive mental stress. However, individuals’ mental responses to the same life event can differ greatly. Therefore, in the present study, mothers were asked not only about whether they experienced various stressful events, such as the above, during early pregnancy; they were also asked to indicate their subjective responses to these stressors. Two tiers of questions were designed to categorize maternal mental stress. The first tier asked if any of the following occurred during early pregnancy: 1) “Did anyone in your family suddenly die or fall severely ill?” 2) “Has there been family conflict?” 3) “Have your relations with colleagues or friends been stressful? 3) “Have you felt a great deal of pressure at work?” 4) “Did you have an accident?”, and 5) “Was a large amount of money stolen from your family?” If the response to one of these questions was affirmative, then a second tier question was asked: Was your mental response severe, mild, or absent? Although the role of maternal stress needs to be validated by additional studies, and the biologic mechanisms by which maternal stress causes CHD require elucidation, we strongly suggest that mental healthcare for pregnant women be augmented, especially during early pregnancy.

The number of previous pregnancies and number of spontaneous abortions were statistically significant in univariate analysis, but only number of previous pregnancies was significant in multivariate analysis (OR, 2.68; 95% CI, 1.44–4.97). Multiparity has been limited in China since the 1980s—when the family planning policy was implemented—which suggests that the association we observed is due to spontaneous abortions. Chinn et al^23^ found that among 400 spontaneous abortuses between 9 and 40 weeks’ gestation, 13.0% had detectable CHD, which suggests that fetuses with CHD often survive to term and that CHD does not significantly affect the survival of the fetus in utero. Because cause and effect cannot be inferred with respect to why increased gestational frequency led to an increase in CHD, these findings suggest that the increase in spontaneous abortions was caused by congenital malformations. Tikkanen et al^24^ observed that a history of several earlier miscarriages was a predictor of an infant born with CHD (OR, 2.7; 95% CI, 1.4–5.3). Ferenca et al^25^ found that a history of miscarriage was associated with an increased risk for tetralogy of Fallot. Therefore, we should strengthen obstetrical care for women who have had spontaneous abortions by implementing regular health counseling and testing to decrease the incidence of pediatric CHD.

TORCH infections—Toxoplasma gondii, Other microorganisms (like syphilis), Rubella virus, Cytomegalovirus, and Herpes viruses—can cause severe birth defects during gestation.^26^ TORCH microorganisms can be easily transmitted to the fetus throughout the pregnancy via the imperfect placental barrier, especially in cases of primary infection during early pregnancy. In 1941, Gregg was the first to describe the relation between maternal rubella virus infection early in gestation and congenital rubella syndrome in offspring.^27^ Since then it has become widely known that maternal microorganism infection during pregnancy can result in the birth of offspring with CHD^28^^–^^31^; hence, much attention has been given to the prevention of TORCH infections in pregnancy. In our study, maternal infection (OR, 7.98; 95% CI, 2.14–29.72) and maternal URI (OR, 4.12; 95% CI, 1.56–10.85) were associated with CHD. The infecting microorganisms that interviewees self-reported included rubella virus, toxoplasma gondii, cytomegalovirus, hepatitis virus, and influenza virus. We did not analyze the influence of each microorganism on CHD because of the limited sample size. URI in the first trimester of pregnancy may be associated with an increased risk for birth defects in offspring. However, these findings should be interpreted cautiously.^32^ Most pregnant women cannot distinguish between a URI and influenza, so it is impossible to eliminate the possibility that influenza may have been misclassified as URI. In addition, Koro'lkova et al^33^ found that Coxsackie B serotypes were associated with CHD. People often take medication for such infections; however, they often cannot recall the drug types and names. Therefore, as compared to collecting information on medication use, it is likely to be easier to ascertain that a participant has a history of infection or URI. Although medications taken during early pregnancy were not analyzed in the multivariate regression model, effects due to drug use cannot be ruled out.

Some studies showed no association between the mother’s education level and CHD^21^^,^^34^; others have found such an association.^35^ We noted that the occurrence of CHD was inversely associated with the mother’s education level (OR, 0.31; 95% CI, 0.15–0.67), and that there was a dose–response relationship between them. We suspect that mothers with a high level of education were better able to care for themselves, to obtain information on reproduction and pregnancy, and to secure access to health service, thereby indirectly reducing the occurrence of CHD. These findings also highlight the need to improve the healthcare and educational opportunities for women of childbearing age, particularly for those with less formal education.

There was no evidence of an association between CHD and maternal exposure to ionizing radiation or video display terminals^24^^,^^25^^,^^36^; however, we did observe an association with B-mode ultrasound examination. It is uncertain whether the maternal B-mode ultrasound examination caused the increasing occurrence of CHD, or if CHD and/or other diseases resulted in the ultrasound examination. We observed that the association was weak for mothers with abnormal findings before the ultrasound examination. We suspect that the association resulted from bias and that the B-mode ultrasound examination was not a cause of CHD, although further study is necessary to resolve this question.

Many epidemiologic studies in China have indicated that CHD is more likely to occur at high altitude than that at sea level. The lower atmospheric oxygen tension present at high altitude was associated with a higher prevalence of CHD—mainly patent ductus arteriosus and atrial septal defects.^37^^–^^40^ Similar results were obtained in South America^41^ and Peru.^42^ The physiological, pathological, pathogenic, and clinical features of heart and pulmonary circulation in related diseases in high-altitude areas were described several decades ago,^43^ but many unanswered questions still exist with respect to causality. Miao et al^44^ ascribed the increase in CHD to postnatal factors, which means that CHD is due to prolonged postnatal hypoxia. We examined whether neonatal asphyxia or hypoxia occurred at the time of birth (OR, 3.74; 95% CI, 1.25–11.18). It is very difficult, but nevertheless important, to determine whether CHD caused neonatal asphyxia or hypoxia, or if neonatal asphyxia or hypoxia resulted in the postnatal occurrence of CHD. We believe the latter is more likely because the Shandong Peninsula is not in a plateau region. If a relation is confirmed, it would indicate that short-term neonatal asphyxia or hypoxia is an important environmental risk factor for CHD occurring after birth.

There were limitations in our study. First, a hospital-based case–control study has inherent weaknesses. Hospital-based cases do not represent the total distribution of CHD occurring in the local population. Usually, cases presenting for treatment in hospitals are individuals with more serious illnesses, more evident symptoms, or higher socioeconomic status. This generally results in some selectivity of hospitalized patients and, because there are various types of CHD, Berkson bias is unavoidable. In addition, it is likely that some potential cases died at a young age and went undetected, and that CHD went undiagnosed in some patients after birth because of the characteristics of the disorder and limitations in diagnosis and treatment. Furthermore, it is difficult to ensure appropriate selection of hospital-based controls. In our study, controls were not randomly selected from all eligible patients in the same medical institutions as the cases; rather, the 2 eligible and available controls whose time of presentation at hospital was closest to that of the case were selected. Although we conducted a matched case–control design, the comparability of cases and controls cannot be guaranteed; however, efforts were made to reduce selection bias. Namely, in the selection of controls, inpatients (84.76%) were preferred; the remaining controls were outpatients (15.24%). We attempted not to select patients who had come for a health checkup or other reasons. If a control were to have undetected CHD, then the apparent OR would be an underestimation of the true risk.

Secondly, we collected information by means of self-reporting, which is easier to implement and requires less manpower, material, and financial resources; however, it can result in recall bias. The time interval between the exposures of interest and the interview, the age, and cultural and educational background of the respondents can all affect the validity of the responses. Fortunately, the matching design, to a certain extent, offsets this exposure misclassification. Recall bias is difficult to control for when the recollection effects between cases and controls differ. Ordinarily, the parents of children with a congenital abnormality are more sensitive to abnormal or adverse pregnancy outcomes. Thus, they may exaggerate or overestimate exposures to harmful factors during pregnancy, even subjectively establishing a false association. For example, when asked, “Did anyone in your family suddenly die or fall severely ill?” cases may consider more distant relatives, or a more distant past, than controls, and may magnify their mental responses to stressful events. The cases may also magnify exposure to radioactive medical examination, microorganism infection or URI, vaccination or drug use, occupational history, and some lifestyle factors (smoking, alcohol consumption, etc.) during and before pregnancy, even if these did not take place before the occurrence of CHD. This would ultimately lead to an overestimation of risk. Masking was used in this investigation, and all interviewers were uniformly trained before the formal interview. Therefore, we believe that investigation bias was minimal in the present study.

Thirdly, the causes of CHD are multifactorial, and the types of CHD are varied and complex. It was therefore impossible, in the present study, to include all the suspected risk factors for CHD. Although some types of CHD have much in common, specific types of CHD result in particular consequences. Moreover, a very large, diverse set of questions on putative risk factors could affect the statistical power of the analysis. To cite one example, in the original data collection for the study, age at pregnancy was broadly classified (<20 years, 20–35 years, and ≥35 years). However, there were relatively few parents aged <20 or ≥35 years. The absence of detail for the 20–35-year-old age group might explain why there was no significant difference in age between cases and controls. In addition, the limited number of age groups may have resulted in a failure to control for confounding by mother’s age at this pregnancy.

Because of these limitations, the findings need to be interpreted cautiously and etiologic association must not be inferred. Nevertheless, this study offers information to assist in developing guidelines for local CHD prevention. The results strongly suggest that: 1) government, society, and families should pay more attention to the mental health of pregnant women, especially during early pregnancy, and should take all practical measures to create a relaxed living and working environment by reducing or eliminating all types of mental stress; 2) women should be offered regular and standardized health counseling and testing during pregnancy—such as TORCH active infection screening during early pregnancy, TORCH series IgM antibody testing, and DNA testing through umbilical cord blood—and should consider vaccination or pregnancy termination, if necessary; 3) women should guard against URI, refrain from taking unnecessary medication in pregnancy, and comply with doctors’ guidance and prescriptions; 4) the government should develop health education materials and communication strategies targeted at women of childbearing age, especially those with less formal education, to enable them to better care for themselves and seek health services; and 5) improve procedures and techniques to guard against neonatal asphyxia or hypoxia.
